# Supplementary figures and images for: Oligo-barcode illuminates holocentric karyotype evolution in Rhynchospora (Cyperaceae)
Source: Front Plant Sci. 2024 Feb 7;15:1330927. doi: 10.3389/fpls.2024.1330927 (PMC10879424; doi:10.3389/fpls.2024.1330927)

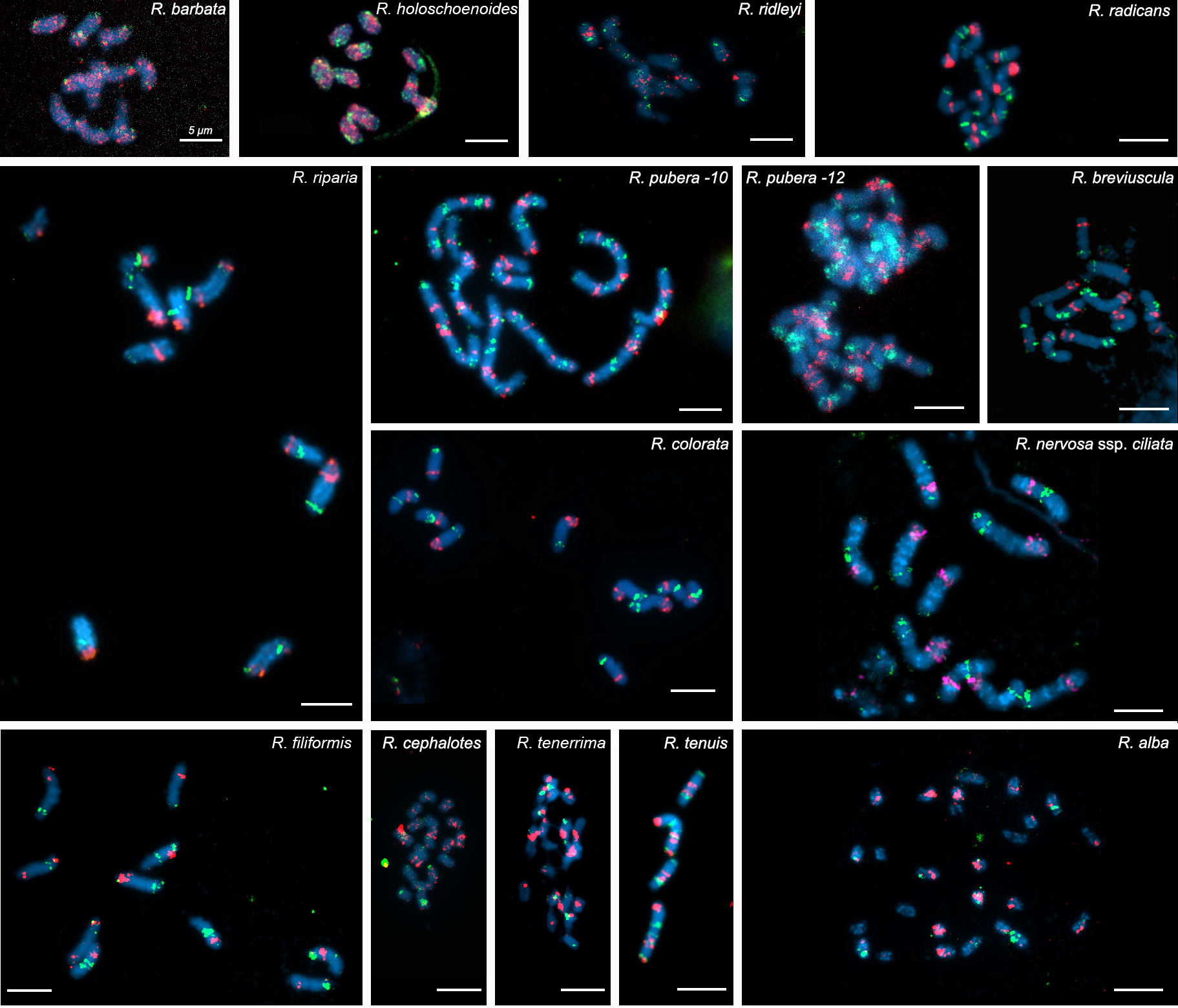

Supplement: Supplementary Figure 1 — Chromosome identification by oligo-FISH barcoding in Rhynchospora species using Rbv-I (green) and Rbv-II (magenta) oligo probes developed based on R. breviuscula (n = 5) reference genome. R. barbata 2n = 10, R. holoschoenoides 2n = 10, R. ridleyi 2n = 12, R. radicans 2n = 10, R. riparia 2n = 10, R. pubera 2n = 10, R. pubera 2n = 12, R. breviuscula 2n = 10, R. colorata 2n = 10, R. nervosa subsp. ciliata 2n = 10, R. filiformis 2n = 10, R. cephalotes 2n = 18, R. tenerrima 2n = 20, R. tenuis 2n = 4, and R. alba 2n = 26. Note that despite some similar patterns to those of R. breviuscula, we could not decipher the complete karyotype of R. pubera-12. Bars = 5 µm. [file Image_1.jpeg]

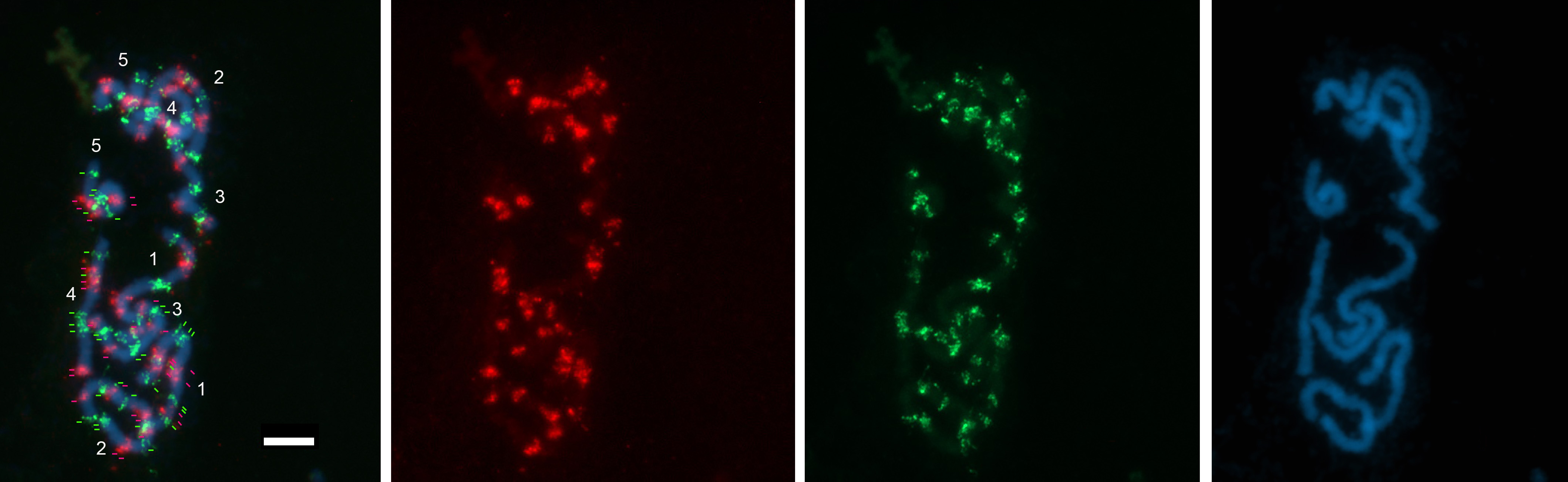

Supplement: Supplementary Figure 2 — Chromosome identification in prometaphase cells by oligo-FISH barcoding in R. pubera-10 using Rbv-I (green) and Rbv-II (magenta) probes developed in R. breviuscula. 8×4 green and 7×4 magenta signals are shown with a total of 60 signals in the haploid genome of R. pubera-10. Bar = 5 µm. [file Image_2.jpeg]
